# High risk of cardiovascular side effects after treatment of Hodgkin’s lymphoma – is there a need for intervention in long-term survivors?

**DOI:** 10.48101/ujms.v126.6117

**Published:** 2021-02-15

**Authors:** Anne Andersson, Gunilla Enblad, Martin Erlanson, Ann-Sofie Johansson, Daniel Molin, Björn Tavelin, Ulf Näslund, Beatrice Melin

**Affiliations:** a Department of Radiation Sciences, Oncology, Umeå University, Umeå, Sweden; b Department of Immunology, Genetics and Pathology, Section Experimental and Clinical Oncology, Uppsala University, Uppsala, Sweden; c Department of Public Health and Clinical Medicine, Umeå University, Umeå, Sweden

**Keywords:** Hodgkin lymphoma, survivorship, cardiovascular side effects, intervention

## Abstract

**Background:**

Hodgkin lymphoma (HL) patients have a good prognosis after adequate treatment. Previous treatment with mantle field irradiation has been accompanied by an increased long-term risk of cardiovascular disease (CVD). This study identified co-morbidity factors for the development of cardiovascular side effects and initiated an intervention study aimed to decrease morbidity and mortality of CVD in HL survivors.

**Design:**

Hodgkin lymphoma patients aged ≤45 years diagnosed between 1965 and 1995 were invited to participate. In total, 453 patients completed a questionnaire that addressed co-morbidity factors and clinical symptoms. Of these, 319 accepted to participate in a structured clinical visit. The statistical analyses compared individuals with CVD with those with no CVD.

**Results:**

Cardiovascular disease was reported by 27.9%. Radiotherapy (odds ratio [OR]: 3.27), hypertension and hypercholesterolemia were shown to be independent risk factors for the development of CVD. The OR for CVD and valve disease in patients who received radiotherapy towards mediastinum was 4.48 and 6.07, respectively. At clinical visits, 42% of the patients were referred for further investigation and 24% of these had a cardiac ultrasound performed due to previously unknown heart murmurs.

**Conclusion:**

Radiotherapy towards mediastinum was an independent risk factor for CVD as well as hypercholesterolemia and hypertension. A reasonable approach as intervention for this cohort of patients is regular monitoring of hypertension and hypercholesterolemia and referral to adequate investigation when cardiac symptoms appear. Broad knowledge about the side effects from radiotherapy in the medical community and well-structured information regarding late side effects to the patients are all reasonable approaches as late effects can occur even 40 years after cancer treatment.

## Introduction

The prognosis for Hodgkin lymphoma (HL) patients is good, with >85% 5-year overall survival as the result of treatment with different modalities, usually chemotherapy and/or radiotherapy, irrespective of gender and stage (1). Until the end of the 1980s, extended field radiotherapy with mantle field, including the upper chest, was commonly used in the treatment of limited-stage HL with prescribed total doses ranging between 38 and 44 Gray (Gy). Chemotherapy was added in more advanced stages (2). The long-term survivors of Hodgkin lymphoma have a risk for long-term side effects such as second malignancies (SM) and cardiovascular disease (CVD), which have been studied in several retrospective studies (3–12). Ten years after treatment, there is an increasing risk in HL survivors for primarily SM and CVD (13). Therefore, for some stages of the disease, radiation doses, field sizes and chemotherapy before radiotherapy have been reduced without affecting the cure rate (14). With an increasing population of cancer survivors, the morbidity and mortality among these individuals have become more obvious (15). Early identification of side effects from previous treatments is needed to reduce the morbidity and mortality in HL long-term survivors. Guidelines for prospective surveillance of this patient group suggest baseline visits and individualized follow-up depending on treatment, but patients as well as physicians seem to lack an awareness of these guidelines (12,16,17). The Swedish Hodgkin Intervention and Prevention study (SHIP) recruited patients from three health care regions in Sweden. These patients were diagnosed with HL at the age of 45 years or younger between 1965 and 1995. This study presents the state of health and identifies the development of CVD in long-term survivors of HL with the intention to prevent morbidity and mortality as the result of long-term side effects in HL survivors.

## Material and methods

Individuals diagnosed with HL at the age of 45 years or younger between 1965 and 1995 were identified using the Swedish Cancer Registry. During these years, mantle field irradiation was the standard treatment for many patients with limited disease. From this cohort, individuals alive at the beginning of 2005 and treated in the Northern Sweden, Uppsala, Örebro and Southern Sweden health care regions were invited to participate in the study. After informed written consent had been obtained, a questionnaire was sent to the patients that included questions concerning HL treatment, if they had any ongoing surveillance program within the health care system, state of health, socioeconomic factors, and family history of both cancer and CVD (i.e. coronary artery disease, congestive heart disease and valvular disease). Since we wanted to use binary logistic regression, individuals who were former smokers were categorized as smokers. No pack-years of smoking were calculated. After returning the completed questionnaire, the patients were offered an open clinic visit. One reminder was sent to the patients who did not return the informed consent and/or questionnaire. The open clinic visit included a thorough medical history with a standardized form of questions targeted to detect the family history of cancer or CVD, symptoms of CVD, clinical investigation, electrocardiogram (ECG) and blood sampling (complete blood count, cholesterol, serum glucose, pro B type natriuretic peptide (pro-BNP) and thyroid-stimulating hormone). Patients with pathological findings in their medical history, at clinical examination or in laboratory test results were referred to a specialist physician for consultation, supplementary investigations, treatment and follow-up. Follow-up (FU) time was set for each patient as the time between the year of HL diagnosis and the year when returning the survey. For the cohort attending open clinic visit or phone visit, follow-up was set as time between year of HL diagnosis and the year of clinical visit. The study was approved by the ethics committee in Umeå (DNR Dnr 05-112M).

### Statistical analyses

The questionnaires were scanned by IT services and system development at Umeå University (ITS) and this was followed by a data cleaning procedure to exclude eventual errors in outliers. Data were analysed using IBM SPSS Statistics, version 23.

A comparison was performed on individuals participating and not participating in the study with Pearson’s chi-squared test to investigate if age at follow-up, gender, region or distance to the hospital were associated with acceptance to the study. Patients were grouped according to young, mid-life and retired status – that is, younger than 40, 40–65 and older than 65 years – to investigate whether there were any systematic differences in age categories. Binary logistic regression analysis was performed on potential covariates – that is, radiotherapy, hypertension, hypercholesterolemia, diabetes, smoking habits and family history of CVD – to investigate their association to the risk for CVD. Smoking habits were categorized into ‘ever smokers’ and ‘never smokers’; former smokers were categorized as ‘ever smokers’. As the covariates are well-known risk factors for CVD, our primary aim was to investigate whether there was an interaction of radiotherapy and other known CVD risk factors for the development of CVD (18). The cumulative risk for CVD, coronary artery disease, valve disease and heart failure was estimated and stratified according to years after HL diagnosis.

## Results

Using the Swedish Cancer Registry, we identified 6,946 individuals diagnosed with HL between 1965 and 1995. At the beginning of 2005, 1,700 of these individuals were living in Sweden. From this cohort, we invited 742 individuals diagnosed at the three participating University Hospitals (Umeå, Uppsala and Lund) at the age of 45 or younger at the time of treatment to participate in the study. From these, 40 (5.4%) were excluded due to death before inclusion (*n* = 24), wrong diagnosis (*n* = 3) or emigrated/no address (*n* = 13). Of the 702 invited individuals, 504 (71.7%) agreed to participate in the study and were mailed a questionnaire; 453 (89.8%) returned a completed questionnaire. These 453 patients had a median follow-up of 22.0 years with a total of 10,796 person-years. Gender, age at invitation to the study and distance to the hospital were not significantly associated with participation in the study (data not shown). From the cohort of 453 HL survivors, 325 (71.7%) individuals accepted the invitation to a clinical visit. After review of the medical records of the clinical visits, six individuals were excluded from the study because their primary HL diagnosis was after the age of 45. Five individuals requested a telephone interview instead of a clinical visit; if applicable, these data were included in the analysis. The remaining 128 individuals declined both a clinic visit and a telephone interview. Characteristics of the 319 individuals included in the final analysis are shown in Table 1.

**Table 1:** Characteristics of the patients attending the clinical visit.

Variable	Male *N* = 164 (51.4)	Female *N* = 155 (48.6)	All *N* = 319
Mean age at diagnosis, year (range)	26 (3–45)	26 (7–43)	26 (3–45)
Mean age at follow up, year (range)	52 (18–85)	50 (22–76)	51 (18–85)
Mean follow up time*, year (range)	25 (12–43)	23 (12–43)	24 (12–43)
CVD, *n* (%)	57 (34.8)	32 (20.6)	89 (27.9)
Eversmoker, *n* (%)	67 (40.9)	55 (35.5)	122 (38.2)
Hypertonia, *n* (%)	41 (25.0)	25 (16.1)	66 (20.7)
Hypercholesterolemia, *n* (%)	46 (28.0)	18 (11.6)	64 (20.1)
Diabetes mellitus, *n* (%)	12 (7.3)	5 (3.2)	17 (5.3)
Family history of CVD, *n* (%)	65 (39.6)	63 (40.6)	128 (40.1)

* Follow-up time calculated from the year of HL diagnosis to year at clinical visit/phone visit.

CVD: cardiovascular diseases.

**Figure 1:**
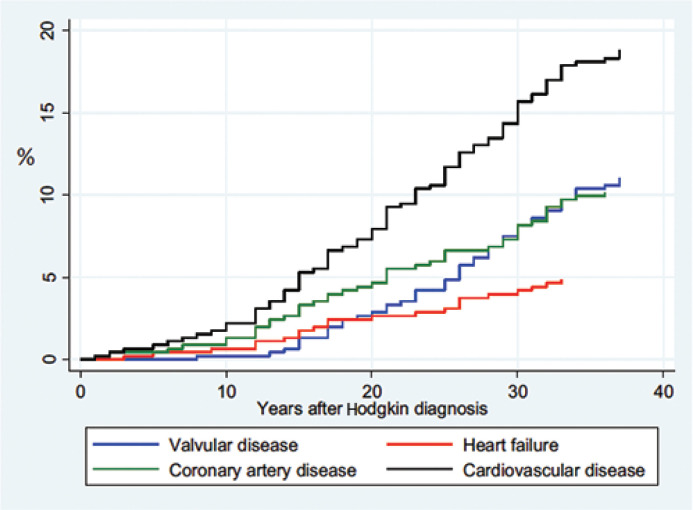
Cumulative incidence of cardiac events. The cumulative incidence of the first event of valve disease, coronary artery disease, heart failure and cardiovascular disease (CVD) overall in 453 Hodgkin lymphoma long-term survivors who developed CVD after treatment of HL (both genders, all ages). Based on data from the questionnaire, another five individuals did not report the year of onset, and that is why they are missing in the graph.

Of the 319 included in the final analysis, 269 (84.3%) received radiotherapy and 89 (27.9%) had CVD at the time of their clinic visit (Table 2). Of the 265 individuals treated with radiotherapy and/or chemotherapy, 82 (31%) had developed CVD; of the 50 individuals treated with only chemotherapy, seven (14%) had developed CVD (*P* < 0.01). The overall cumulative incidences of CVD and of different CVD are detailed in Figure 1. Of the 219 individuals who had irradiation towards the mediastinum and who attended the clinic visits, 92 (41.6% of irradiated patients) were referred for further investigation, either cardiac ultrasound (*n* = 31) and/or internal medicine/cardiology consultation as the result of clinical or laboratory findings (*n* = 63). The overall incidence of CVD in the HL cohort increased continuously after treatment and this increase accelerated after 10 years. Heart failure and coronary artery disease showed a stable increase during follow-up. The development of valve dysfunction accelerated 10 years or longer after HL diagnosis. The mean age of the first CVD onset was 51 years and the mean latency time from HL diagnosis to CVD was 26 years.

**Table 2:** Treatment characteristics of patients at an open clinic visit.

Variable	Male	Female	All
*N* (%)	164 (51.4)	155 (48.6)	319
Chemotherapy, *n* (%)	84 (51.2)	91 (58.7)	175 (54.9)
Radiotherapy, *n* (%)	133 (81.1)	136 (87.7)	269 (84.3)
Towards mediastinum*, *n* (%)	103 (77.4)	116 (85.3)	219 (81.4)
Mantle field*, *n* (%)	64 (48.1)	79 (58.1)	143 (53.2)
Radiotherapy + chemotherapy, *n* (%)	56 (34.1)	69 (44.5)	125 (39.2)
Only radiotherapy, *n* (%)	80 (48.8)	64 (41.3)	144 (45.1)
Only chemotherapy, *n* (%)	28 (17.1)	22 (14.2)	50 (15.7)
Dose ≥ 40 Gy	91 (55.5)	85 (54.8)	176 (55.2)

* Percent of individuals who received radiotherapy towards mediastinum and mantle field is calculated among individuals who received radiotherapy.

Binary logistic regression analyses were performed in the cohort of cases with CVD compared with those without CVD. In a univariate model, a significant association was identified with mediastinal radiotherapy (odds ratio [OR]: 4.48, 95% confidence interval [CI]: 2.26–8.88), hypercholesterolemia (OR: 8.45, 95% CI: 4.61–15.48) and hypertension (OR: 3.85, 95% CI: 2.17–6.75). The multivariate model revealed mediastinal radiotherapy, hypercholesterolemia and hypertension as independent risk factors (Table 3). The questionnaire answers revealed 58 individuals who reported valvular disease. Radiotherapy, especially for mediastinum and hypercholesterolemia, was independently associated with a risk for valve disease (OR: 6.14, 95% CI: 2.30–16.40 and OR: 3.40, 95% CI: 1.70–6.78, respectively). Finally, radiotherapy for mediastinum was an independent risk factor for the development of coronary artery disease (OR: 4.41, 95% CI: 1.54–12.7) (Table 2).

**Table 3:** Univariate and multivariate regression analysis.

Variable	Cardiovascular disease* (*n* = 89)				Valve disease (*n* = 58)				Coronary artery disease^†^ (*n* = 46)			
	Univariate logistic regression analysis		Multivariate logistic regression analysis		Univariate logistic regression analysis		Multivariate logistic regression analysis		Univariate logistic regression analysis		Multivariate logistic regression analysis	
	OR	CI (95%)	OR	CI (95%)	OR	CI (95%)	OR	CI (95%)	OR	CI (95%)	OR	CI (95%)
Radiotherapy (*n* = 265)^‡^	3.27	1.34–7.98	-	-	13.175	1.78–97.5	-	-	1.62	0.61–4.32	-	-
Radiotherapy towards mediastinum (*n* = 219)	4.48	2.26–8.88	5.41	2.49–11.76	6.07	2.34–15.70	6.14	2.30–16.40	3.50	1.43–8.56	4.42	1.54–12.67
Hypertensio*n* (*n* = 66)	3.85	2.17–6.75	2.61	1.33–5.14	3.01	1.62–5.61	2.10	1.03–4.28	4.88	2.63–9.04	2.36	1.05–5.30
Hypercholesterolemia (*n* = 64)	8.45	4.61–15.48	7.25	3.69–14.24	4.31	2.32–8.03	3.40	1.70–6.78	16.39	8.25–32.59	13.35	6.15–28.99
Family history of CVD (*n* = 128)	1.32	0.80–2.16	1.12	0.62–2.00	0.89	0.50–1.60	0.76	0.40–1.44	2.64	1.44–4.84	1.20	0.55–2.60
Diabetes (*n* = 17)	1.88	0.69–5.10	1.27	0.35–4.63	1.96	0.66–5.79	1.61	0.44–5.98	5.06	2.02–12.65	3.92	0.98–15.67
Eversmoker (122)	1.30	0.79–2.13	1.30	0.72–2.33	0.98	0.55–1.77	1.03	0.54–1.95	1.41	0.77–2.55	1.13	0.52–2.46

Note: Univariate and multivariate regression analysis on CVD, valve disease and coronary artery disease compared to cases with no CVD, both gender and all ages. In the multivariate analysis, all variables in the table are included, except radiotherapy.

* Cardiovascular disease (CVD) includes coronary artery disease, heart failure and valve disease.

† Coronary artery disease (CAD) includes myocardial infarction.

‡ Radiotherapy (unspecified target) is not included in the multivariate model in favour of radiotherapy towards mediastinum.

CI: confidence interval; OR: odds ratio.

Treatment characteristics are listed in Table 3. From the initial study cohort of 6,946 HL patients, 2,462 died of causes other than HL. Of these, 640 (26%) died of heart disease before we initiated the study, with a median time of 6 years from HL diagnosis to death of heart disease. In our living cohort, 44% (319/702) participated in a clinical visit. There were no obvious systematic differences between the invited and the final group for age, gender or geographical area (data not shown). We did not have ethical permission to check medical records for side effects in patients who for some reason did not participate.

## Discussion

In this study with a long median FU time from diagnosis of HL to entering the study (22 years), mediastinal radiotherapy was shown to be an independent risk factor for CVD in HL long-term survivors, especially for the development of valvular disease. In this cohort, the well-known risk factors for CVD, hypercholesterolemia and hypertension were also independent risk factors for developing CVD. Surprisingly, other risk factors such as smoking and family history of CVD did not increase the risk of CVD; however, the incidence of CVD developed continuously from the end of treatment.

A retrospective Dutch study of 1,474 HL survivors treated at 40 years or younger between 1965 and 1995 found that 31% were deceased, but the study had a slightly shorter follow-up time than our study (13). The Dutch study collected treatment-related data and data concerning risk factors from medical records, questionnaires, general practitioners or attending physicians: 84% received radiotherapy towards the mediastinum alone or in combination with chemotherapy and the frequency of heart failure was 13.3% for patients receiving more than 21 Gy to the heart. Thus, data from our study are in line with those in that report, although the frequency of multiple CVD was higher in the Dutch study compared with our study (44.4% vs. 31.5%) (3,17). This difference could be explained by the fact that our study included only prospective cases living at the time when our surveillance study started. Valve disease and coronary artery disease were the most frequent diagnoses as in the present investigation. In a study of 82 HL survivors who underwent screening for valvular disease (19), the mantle-irradiated individuals had a significantly increased risk for valvular dysfunction compared with those receiving chemotherapy. As in our study, this risk increased after 10 years, suggesting that surveillance with echocardiography 10 years after treatment with radiotherapy towards mediastinum should be carried out.

A Norwegian group presented an echocardiography study of 116 HL survivors treated at the age of <50 years and with a follow-up of median 5 years. They found aortic and mitral valve regurgitation grade >1 in 24% of the patients and therefore suggested echocardiography screening in HL survivors at risk (20). More recent studies have shown that these valve defects over time develop into clinically significant valve disease demanding surgical intervention. Current recommendations for childhood cancer survivors who received radiation to the heart exceeding 20 Gy include echocardiography and cardiac exercise test once every 5 years during adulthood.

We propose a similar approach for adult HL survivors having received radiotherapy to the mediastinum before the age of 45 years (21). An alternative to regular echocardiograms could be regular cardiac auscultation and referral to echocardiography for the individuals presenting with a heart murmur. In our study, 25% of the individuals examined at the offered clinical visit needed referral due to previously unknown heart murmur. Economic considerations might play a role in the choice between regular echocardiograms and regular heart auscultation.

Our study and the Dutch study included data on other risk factors such as smoking habits, hypertension, hypercholesterolemia and family history. In both studies, hypercholesterolemia was shown to affect the development of valve disease and coronary artery disease in HL survivors, obvious targets for intervention. In a hypothetical cohort of HL long-term survivors treated at 30 years of age and with a 5-year follow-up after mediastinal irradiation, Chen et al. found that lipid screening every 3 years was cost-effective and statin treatment for individuals with elevated lipid levels improved survival (22). Therefore, patients receiving mediastinal radiotherapy should receive active secondary prevention treatments, blood pressure monitoring and cholesterol testing at a follow-up to lower incidence and mortality of CVD. The benefit from primary prevention of known risk factors of CVD has been shown in a study of 1893 individuals in a community in Northern Sweden; specifically, the study found that a long-term community-based CVD prevention program was associated with a decreased risk for death from CVD by 26.1% (23). Since the frequency of CVD in HL survivors greatly exceeds the frequency in healthy individuals, targeted intense intervention ought to be cost-effective even though no health economic studies have been conducted that focus on HL survivors (24).

As there is a long latency for CVD as a late complication of HL treatment and assessing cardiovascular morbidity are of great importance, screening and surveillance of HL long-term survivors at high risk of severe CVD need to be considered, a proposal that has been made by earlier studies. For example, Van Leeuven et al. suggested screening every 5 years starting 5 years after mediastinal radiotherapy in individuals at high risk for CVD and after 10 years in other HL survivors (25). Suggested screening methods include CAC-score (coronary artery calcium score), computed tomography (CT)-angiography, echocardiography, ECG and monitoring of risk factors for CVD. The SHIP study recommends prospective clinical visits at regular intervals to detect new heart murmurs, to monitor other risk factors such as hypertension and hypercholesterolemia, and an echocardiography every 5 years could be suggested rather than just a follow-up. We consider these recommendations to be a reasonable intervention level to detect independent risk factors with the aim to lower mortality rates in patients given mediastinal radiotherapy.

### Limitations

A limitation of this study is that we had a drop-out of approximately 28%. However, because the participants had been treated a long time ago, this drop-out rate is acceptable as some patients would likely want to avoid conjuring up their memories of treatment. We also did not include the diseased patients as the major aim was to put living individuals on a surveillance scheme. The cohort size limited the possibility to find moderately strong associations.

### Conclusion

Modern treatment for HL has abandoned the use of extensive field radiotherapy in favour of chemotherapy and new irradiation techniques that lower the risk of long-term side effects from mediastinal irradiation. Nevertheless, a number of patients need irradiation to the mediastinum and these individuals will also benefit from surveillance programs developed for the cohorts of patients in the present study.
